# Seasonal Dietary Patterns of Tarim Red Deer (*Cervus hanglu yarkandensis*) Revealed by the *trnL* Sequencing Approach in the Tarim River Basin (Xinjiang, China)

**DOI:** 10.1002/ece3.72757

**Published:** 2025-12-23

**Authors:** Cheng Qi, Xiangzhou Tian, Yuanfan Cui, Linyin Zhu, Stefano Focardi, Linqiang Zhong

**Affiliations:** ^1^ Xinjiang Key Laboratory of Biological Resources and Genetic Engineering, College of Life Science and Technology Xinjiang University Urumqi China; ^2^ Istituto Dei Sistemi Complessi del CNR Sesto Fiorentino FI Italy

**Keywords:** *Cervus hanglu yarkandensis*, dietary habits, DNA metabarcoding technology, mixed feeder

## Abstract

The Tarim red deer (*Cervus hanglu yarkandensis*) is a flagship species inhabiting the Euphrates poplar (*Populus euphratica*) riparian forests along the Tarim River in China. It is listed as a Class I nationally protected species but despite its importance, current knowledge about this subspecies remains limited, particularly regarding the seasonal variation in its foraging ecology within the Shaya region of Xinjiang—a landscape characterized by a mosaic of Euphrates poplar forests interspersed with cotton ( *Gossypium hirsutum* ) croplands. We used DNA metabarcoding technology to investigate the diet of the Tarim red deer during the summer and winter of 2021–2022. We targeted chloroplast gene *trnL* (c–h) region for plant identification. From a total of 39 fecal samples analyzed, we identified 50 plant species; based on the frequency of occurrence, the primary food items of Tarim red deer were cotton (38.75%) *and* Euphrates poplar (36.87%). Other species were consumed occasionally, including common reed ( *Phragmites australis* , 7.00%), common salt tree ( *Halimodendron halodendron* , 6.13%), goosefoot ( *Chenopodium album* , 4.67%), licorice (*Glycyrrhiza inflata*, 3.50%), slender branch saltwort (*Kalidium gracile*, 0.28%), Chinese licorice ( *Glycyrrhiza glabra* , 0.25%), elegant false tamarisk (*Myrtama elegans*, 0.16%), and Oriental dodartia (*Dodartia orientalis*, 0.07%). As expected, both the Shannon diversity index and species richness were significantly higher in summer than in winter (*p* < 0.05), whereas the dietary niche breadth was significantly lower in winter (*p* < 0.05). Overall, our results indicate that the Tarim red deer is a seasonally adaptable mixed feeder. This study provides valuable insights into the foraging ecology of this endangered subspecies and offers scientific evidence to support its management and conservation. In particular, we stress the importance of cotton in their diet and stressed that measures of mitigation could affect the conservation of this endangered population.

## Graphical abstract

This study employed DNA metabarcoding technology to analyze the seasonal dietary habits of Tarim red deer in Xinjiang within a mixed habitat of *Populus euphratica* and cotton. Results indicate a primary reliance on cotton (38.75%) and European poplar (36.87%). Summer dietary diversity and ecological niche breadth were significantly higher than in winter (*p* < 0.05), confirming their adaptive role as seasonally flexible omnivores. This finding provides crucial ecological evidence for developing targeted conservation measures, holding significant ecological value for this protected species.

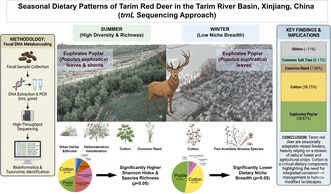

## 1 Introduction

The Tarim River Basin is an arid area featuring fragmented vegetation cover and an ecosystem highly susceptible to degradation (Shao et al. 2016). Since the 1960s, the quality of wildlife habitat in this region has been deteriorating due to human population growth, human activities, socioeconomic development, and uncontrolled exploitation of water resources (Anwar and Halik 2008; Yang et al. 2025). Therefore, the combination of ecological degradation and unique biodiversity is the reason for the basin's designation as one of China's 35 priority areas for biodiversity protection (Protection M. o. E 2011). The implementation of two decades of integrated management and regulated water flows in the Tarim River basin has led to the preservation and partial rehabilitation of the Euphrates poplar (*Populus euphratica*) ecosystem in its middle and lower reaches (Jiudan et al. 2022; Zhang et al. 2022; Wang, Hu, et al. 2023; Wang, Li, et al. 2023; Wang, Zhao, et al. 2023).

The Tarim red deer (*Cervus hanglu yarkandensis*), a large ungulate with high adaptability to arid habitats, is essential for preserving the functional integrity of arid and semi‐arid forest ecosystems and the conservation of regional biodiversity (Tumur et al. 2013; Ababaikeri et al. 2020; Ba et al. 2020). The Tarim red deer population is currently distributed only in the Tarim and Cherchen river basins and appears to be fragmented in three distinct sub‐populations: Shaya, Lopnur, and Qarqan. It has declined to approximately 450 individuals and has undergone severe genetic bottlenecks, resulting in markedly reduced genetic diversity (Mahmut et al. 2001, 2002). The habitat of the Tarim red deer now exists as a mosaic of fragmented Euphrates poplar forests and cotton fields, resulting from agricultural expansion (Halik et al. 2004; Dong et al. 2010).

Recent phylogenetic studies have not only established the Tarim red deer as a distinct subspecies of the hangul (*Cervus hanglu*)—a taxon widely distributed in Central Asia along the Amu Darya River and in Kashmir, but it has been shown that the Tarim red deer has accumulated a large portion of deleterious substitutions in genes coding for mitochondrial protein, probably caused by decreasing population size (Mackiewicz et al. 2022; Ahmad et al. 2025). Accordingly, Tarim red deer is classified as an endangered species by the IUCN and listed as a Category I protected animal in China (Tumur et al. 2013). In recent years, research on the Tarim red deer has primarily focused on population size and distribution, habitat selection (Abliz et al. 2010; Mamamt et al. 2018), ecological characteristics (Tayerjan et al. 2021), and conservation genetics (Ababaikeri et al. 2020, Buweihailiqiemu et al. 2023). However, research on the feeding ecology of the Tarim red deer within habitat mosaics characterized by interspersed poplar forests and cotton fields is still relatively scarce.

Diet composition serves as the primary functional interface between an animal and its habitat. It fulfills the essential role of supplying energy and nutrients, while simultaneously shaping the trophic ecological niche of the species (Wang et al. 2025). Consequently, the analysis of diet composition constitutes a foundational component of ungulate nutritional ecology. It is instrumental for determining food requirements, thereby elucidating habitat selection patterns and providing critical insights for the conservation management of endangered species (Van Soest 2018; Wang, Hu, et al. 2023; Wang, Li, et al. 2023; Wang, Zhao, et al. 2023).

Contemporary dietary analysis for herbivores employs a suite of techniques, encompassing behavioral observation, utilization surveys, microscopic analysis of feces, examination of stomach contents, near‐infrared reflectance spectroscopy (Walker et al. 2002; Beringer et al. 2004; Si et al. 2007; Scasta et al. 2016), with DNA metabarcoding technology emerging as a powerful tool due to advances in high‐throughput sequencing (Garnick et al. 2018). DNA metabarcoding employs standardized genetic markers to enable high‐throughput species identification from complex environmental samples. Contrary to the common ecological assumption that larger body size enables broader resource use, DNA metabarcoding analysis of seven large mammalian herbivores revealed that dietary niche breadth did not increase significantly with body size (Kartzinel et al. 2015). The application of DNA metabarcoding to the dietary analysis of Korean water deer ( *Hydropotes inermis* ) revealed the consumption of plant families including Pinaceae, Araliaceae, Potamogetonaceae, and Apiaceae, which had remained undetected by conventional methodological approaches (Lee et al. 2022). Dietary analysis revealed a significant dietary overlap between sika deer ( *Cervus nippon* ) and Reeves's muntjac ( *Muntiacus reevesi* ), while both species exhibited substantially different foraging patterns from the Tolai hare ( *Lepus tolai* ) (Wang, Hu, et al. 2023; Wang, Li, et al. 2023; Wang, Zhao, et al. 2023). Thus, accumulated empirical evidence firmly establishes that non‐invasive fecal DNA metabarcoding has evolved into a well‐validated and reliable methodology for characterizing the dietary ecology of large mammals (Ando et al. 2020).

Noteworthy, a main concern for the conservation of the Tarim deer is represented by the conflict with cotton farmers. It is important for managers to evaluate the impact of dissuasion (for instance the use of fences) on the foraging ecology of the Tarim deer. Another important parameter for the conservation of the Tarim red deer in its fragile habitat is represented by the assessment of the nutritional carrying capacity of the poplar ecosystem whose nutritional value for deer is probably impacted by the presence of domestic livestock in our study area (Hobbs and Swift 1985). The computation of nutritional carrying capacity requests precise information about the diet of deer in different seasons. In particular, the foraging ecology of the Tarim deer should be evaluated. In order to address the conservation issues described above, we collected fecal samples and used the DNA barcoding technique to determine diet composition as a function of season. Our study provides a first comprehensive understanding of the forage use of the Tarim red deer, which will provide basic information very useful for its conservation and habitat management in the Tarim River Basin of Xinjiang.

In short, we aim to provide a first assessment of dietary patterns of Tarim deer in the season of plenty (summer) and in the most critical period for the species (winter) when temperature is always below zero degree for several months given the climatic conditions of the study area, we expect to observe a strong seasonal pattern in resource use, evaluate the importance of use of cotton because this is the critical element which determine human–deer conflicts in the area, we wish to assess inter‐individual variability in resource use in order to understand at which extent cotton is a substitutable resource for Tarim red deer.

We hypothesized that (i) dietary diversity would be higher in summer than in winter due to greater food availability, (ii) Euphrates Poplar and shrub species would dominate the diet throughout the year as an adaptation to arid riparian habitats, and (iii) cotton constitutes a key dietary resource for Tarim red deer during periods of natural forage limitation in winter, thereby intensifying human–deer conflicts. This study provides essential baseline data for understanding the feeding ecology of Tarim red deer and offers insights to guide habitat management and conservation strategies for large herbivores in arid ecosystems.

## 2 Materials and Methods

### 2.1 Study Area and Sample Collection

**FIGURE 1:**
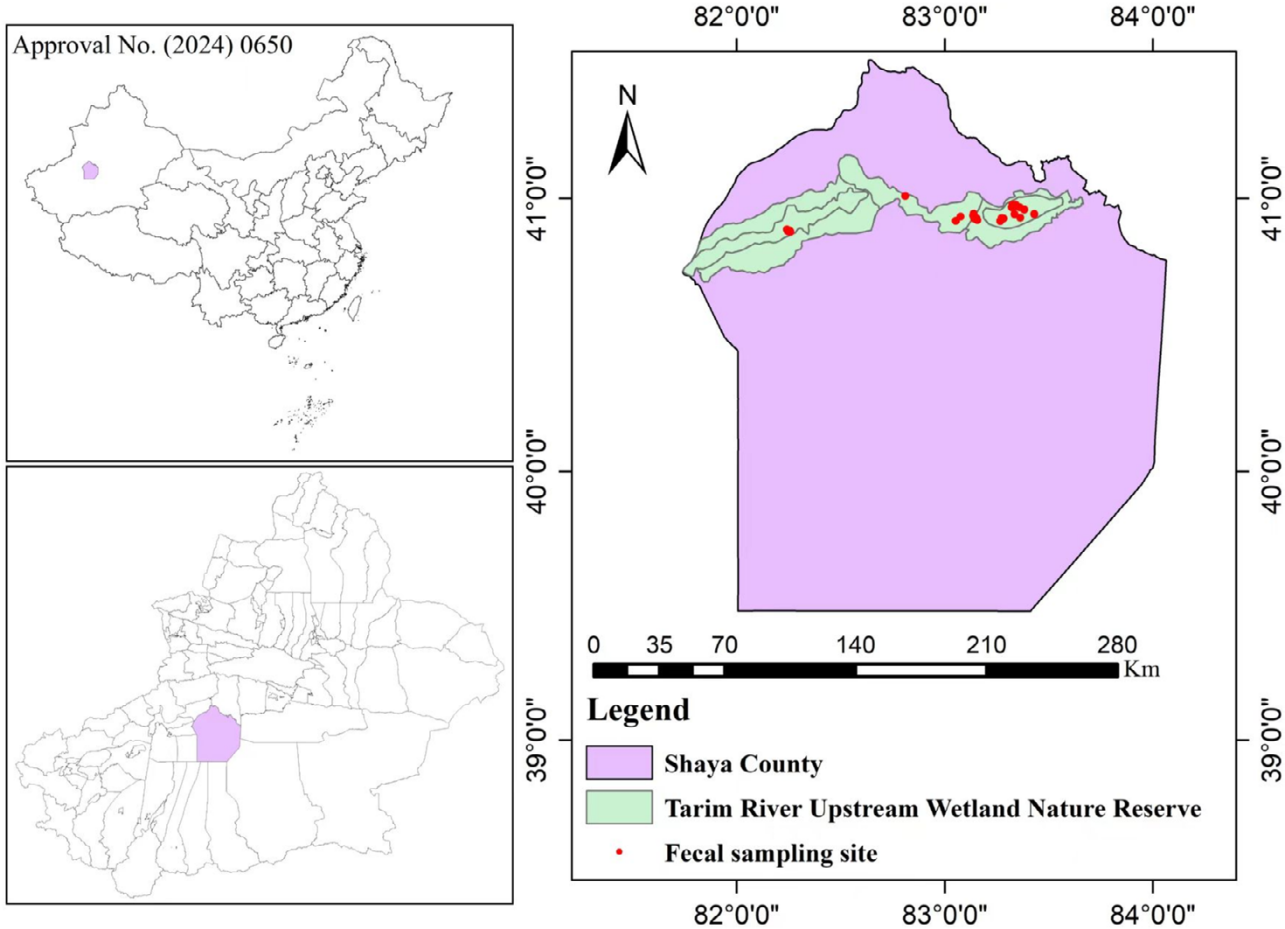
Geographic locations of fecal samples of Tarim red deer in summer and winter.

The research was conducted in the Tarim River Upper Wetland Nature Reserve, located in Shaya County, in the northern region of the Tarim Basin, Xinjiang, China (Figure 1), during the summer (July) 2021 and winter (January) 2022. This reserve constitutes the core distribution area of the Tarim red deer population.

The reserve (39°31′–41°25′N, 81°45′–84°47′E), covering a total area of 25,6840.0 km^2^, belongs to the temperate continental arid climate and is characterized by high sunshine intensity, low precipitation, dry weather, and significant temperature variation between day and night. The primary vegetation of the study area consists of poplar forests, tamarisk bush ( *Tamarix ramosissima* ), and salinization grassland, including licorice (*Glycyrrhiza inflata*), common salt tree ( *Halimodendron halodendron* ), camelthorn (*Alhagi sparsifolia*), and common reed ( *Phragmites australis* ) (Zhu et al. 2015). The reserve accommodates a range of protected wildlife, such as Goitered gazelle ( *Gazella subgutturosa yarkandensis* ), the Yarkand hare ( *Lepus yarkandensis* ), and red fox ( *Vulpes vulpes* ).

A total of 58 fecal samples were collected during the summer (28 samples) and winter (30 samples) from the main activity areas of Tarim red deer. To ensure a diverse range of sample sources, we collected samples from many vegetation types and in the known distribution areas of the target animal within the reserve (Figure 1).

### 2.2 Individual Identification

The fecal pellets were scraped from the outer surface to collect the relic intestinal epithelial cells, and genomic DNA was extracted from fecal pellets and muscle samples of Tarim red deer using the QIAamp Fast DNA Stool Mini Kit (QIAGEN company) and the TIANamp Genomic DNA Kit (Axygen Biosciences); the muscle samples were obtained from roadkill carcasses collected earlier by the research team. We stored the extracted DNA at −80°C until PCR amplification. Then, we amplified fecal DNA samples through PCR using five pairs of microsatellite primers (Mahmut et al. 2001; Table 1) and sent the PCR products to Bioasia Biotechnological Company (Shanghai) for STR (short tandem repeats) typing. Based on the results of microsatellite DNA testing, we used Cevus 3.0 software (https://cervus.software.informer.com/3.0/) for individual identification analysis (Qianqian et al. 2013).

**TABLE 1:** PCR primer sequences for sexual and individual identification.

Locus	PCR primer (5′→3′)	*T*_m_ (°C)
BM888‐F	AGGCCATATAGGAGGCAAGCTT	49
BM888‐R	CTCGGTCAGCTCAAAACGAG	
BM4208‐F	TCAGTACACTGGCCACCATG	56
BM4208‐R	CACTGCATGCTTTTCCAAAC	
BM5004‐F	TCTGGAGTGAATGTTTCTGAGG	49
BM5004‐R	TTGTGATGAGCACCTGAAGG	
BM6438‐F	TTGAGCACAGACACAGACTGG	58
BM6438‐R	ACTGAATGCCTCCTTTGTGC	
BM6506‐F	GCACGTGGTAAAGAGATGGC	63
BM6506‐R	AGCAACTTGAGCATGGCAC	
SRY12‐F	CTTCATTGTGTGGTCTCGTG	61
SRY12‐R	CGGGTATTTGTCTCGGTGTA	
BMC1009‐F	GCACCAGCAGAGGAGGACATT	52
BMC1009‐R	ACCGGCTATTGTCCATCTTG	

### 2.3 Reference Databases Construcation

Based on the plant lists of the species identified in the study area, plant classification and scientific names were adjusted using the Biodiversity Committee of the Chinese Academy of Sciences, 2023 (http://www.sp2000.org.cn/CoLChina). The chloroplast *trnL* gene sequences of each plant species were downloaded from NCBI (https://www.ncbi.nlm.nih.gov/) to establish a DNA barcode reference database of plant species of the study area. The database consists of 3 phyla, 45 families, 138 genera, and 294 species of *trnL* gene sequences and was created by Shanghai Lingen Biological Company (http://www.biozeron.com/).

### 2.4 DNA Extraction From Fecal Food Residues Sequencing and Sequencing

Total DNA was extracted from Tarim red deer fecal samples according to the instructions of the E.Z.N.A. Soil DNA Kit (Omega Bio‐tek, Norcross, GA, USA). The chloroplast gene‐specific primers *trnL*‐c (5′‐GGGCAATCCTGAGCCAA‐3′) and *trnL*‐h (5′‐CCATTGAGTCTCTGCACCTATC‐3′) were utilized for the amplification and sequencing of fecal DNA samples from Tarim red deer. Each 20 μL PCR mixture was comprised of 4 μL of 5× FastPfu Buffer, 2 μL of 2.5 mM dNTPs, 0.8 μL (5 μM) of each primer, 0.4 μL of FastPfu Polymerase, and 10 ng of DNA extract, and supplemented with ddH_2_O to 20 μL. The PCR amplification reaction procedure was as follows: 2 min at 95°C for initial denaturation; 35 cycles of 30 s at 95°C, 30 s at 55°C, 1 min at 72°C, and 5 min at 72°C final extension. The PCR products were subjected to 2% agarose gel electrophoresis for size separation. Subsequently, the DNA bands of interest were purified using the AxyPrep DNA Gel Extraction Kit (Axygen Biosciences, Union City, CA, USA), following the manufacturer's protocol. Based on the preliminary quantitative analysis from agarose gel electrophoresis, the PCR products were detected and quantified using the QuantiFluor‐ST Blue Fluorometric Quantitation System (Promega). Following quantification, the PCR products were mixed in ratios appropriate for the sequencing yield requirements of each sample. Subsequently, sequencing libraries were constructed, and the samples were sequenced on the Illumina PE250 platform.

### 2.5 Data Analysis

The paired‐end (PE) reads of the *trnL* gene were aligned based on base overlap relationships, and simultaneous quality control and filtering were conducted to generate high‐quality clean reads. First, valid sequences for all samples are obtained based on the barcode. Then, quality control and filtering are performed on the reads. Subsequently, paired‐end reads are merged into a single sequence utilizing the overlap between them. Finally, demultiplexing is conducted according to the barcode and primer sequences to acquire high‐quality sequences for each sample, during which the sequence orientation is adjusted based on the forward and reverse barcodes and primer directions, and chimeras are removed. The specific parameters are as follows:

1. Reads are filtered by truncating bases from the tail with quality scores below 20. A sliding window of 10 bp is applied; if the average quality value within the window is below 20, the sequence is truncated from the start of the window. Reads shorter than 50 bp after quality control are discarded.
2. Paired‐end reads are merged into a single sequence based on the overlap between them, with a minimum overlap length of 10 bp.
3. For the merged sequences, the maximum allowed mismatch ratio in the overlap region is 0.2, and non‐compliant sequences are filtered out.
4. Samples are distinguished based on the barcode and primer sequences at both ends of the sequences, and the sequence orientation is adjusted. No mismatches are allowed for barcodes, while a maximum of 2 mismatches is permitted for primers.
5. Chimeras are removed using Usearch software and the gold database, employing a hybrid approach that combines de novo and reference‐based methods.

These sequences (reads) were then clustered into operational taxonomic units (OTUs) with a 97% similarity threshold. The OTUs sequences were compared against a curated reference database of *trnL* gene sequences from plants in the protected area using BLAST+ (version 2.3.0). Taxonomic assignments were made for each OTUs sequence based on sequence coverage and identity of at least 97%. When a sequence matches two or more taxonomic units, the sequence is assigned to a higher‐level taxonomic unit. Based on the OTUs sequence clustering results, packages such as vegan, ggplot2, ggsignif, indicspecies, and ggforce and RColorBrewer in R (R‐4.2.3) are used to calculate Richness index and Shannon index.

The rarefaction curve, which serves as a graphical representation of the relationship between sampling effort and the number of observed species, is generated using the rarefaction.single command of Mothur software and is based on the Shannon–Wiener index and richness index (http://www.cloud.biomicroclass.com/CloudPlatform/SoftPage/RFC). Furthermore, a stacked plot depicting the taxonomic composition at the food species level is constructed using OTUs clustering data with the ggplot2 package in R.

Indicator species analysis was performed to identify taxa sensitive to environmental changes and capable of reflecting specific ecological conditions. This analysis was conducted on the cloud‐based bioinformatics platform provided by MajorBio (https://cloud.majorbio.com/page/tools.html).

We conducted β‐diversity analyses to investigate the differences in diet composition across seasons. Specifically, we visualized the compositional differences between the samples using non‐metric multidimensional scaling (NMDS) based on the Bray–Curtis dissimilarity, employing the metaMDS function. We used PERMANOVA (Permutational Multivariate Analysis of Variance) to compare groups based on multivariate data without relying on assumptions of normality or homogeneity of variances and the ANOSIM test to measure the degree of between‐groups separation (Clarke 1993; Anderson 2010). Additionally, we calculated niche width between seasons using Levins' test from the spaa package. The normality of the data was assessed using the base package, while the test for homogeneity of variances was performed with the car package. Finally, the difference test was executed using the stat package in R (R‐4.2.3). The code for data analysis can be found in Supporting Information (R scripts).

## 3 Results

### 3.1 Sequencing Results

**FIGURE 2:**
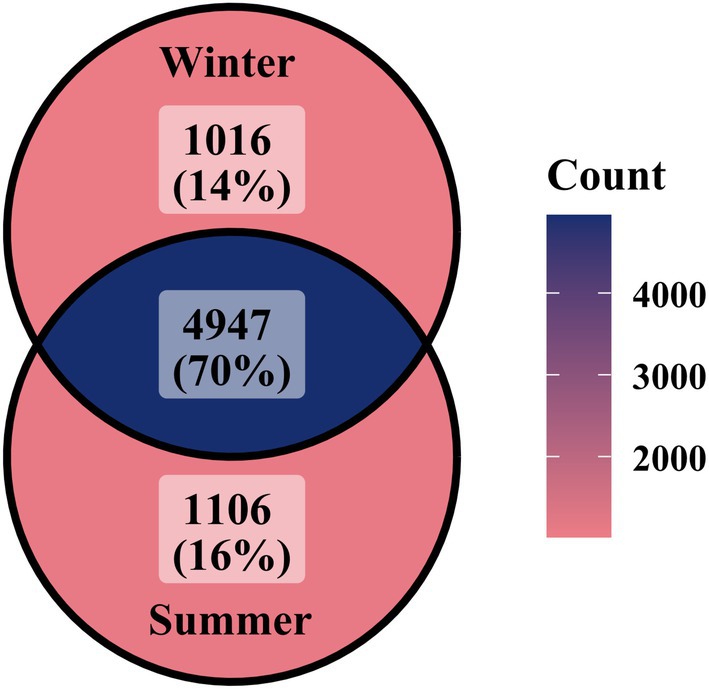
Venn diagram showing the number of shared Operational Taxonomic Units.

17 fecal samples failed to amplify microsatellites due to poor DNA quality, while two additional samples were excluded after matching them to two distinct individuals already represented in the summer sample cohort. Through individual identification analysis, we screened 39 fecal samples of Tarim red deer from different individuals, comprising 25 samples collected in winter and 14 collected in summer. High‐throughput sequencing analysis was performed on 39 fecal samples from Tarim red deer employing DNA metabarcoding techniques, resulting in the generation of 24,754,108 high‐quality clean reads. The sequencing depth per sample averaged 634,720 reads, with an average read length of 165 base pairs. Subsequent OTUs picking, based on a 97% sequence similarity threshold, yielded a total of 7095 distinct OTUs. The Shannon index and richness index based dilution curve analysis revealed that with increasing sequencing depth, the dietary diversity did not exhibit significant changes, and the curve remained stable (Figures S1 and S2). This stability suggests that the sequencing depth was sufficient to capture the true diversity of the sample, thereby validating the reliability of the sequencing results and justifying the proceeding to downstream analyses. The Venn chart, constructed from OTUs data, revealed that a total of 4947 (70%) OTUs were shared among the 39 samples, indicating a significant overlap in the dietary composition of Tarim red deer across summer and winter (Figure 2).

### 3.2 Diet Composition

**FIGURE 3:**
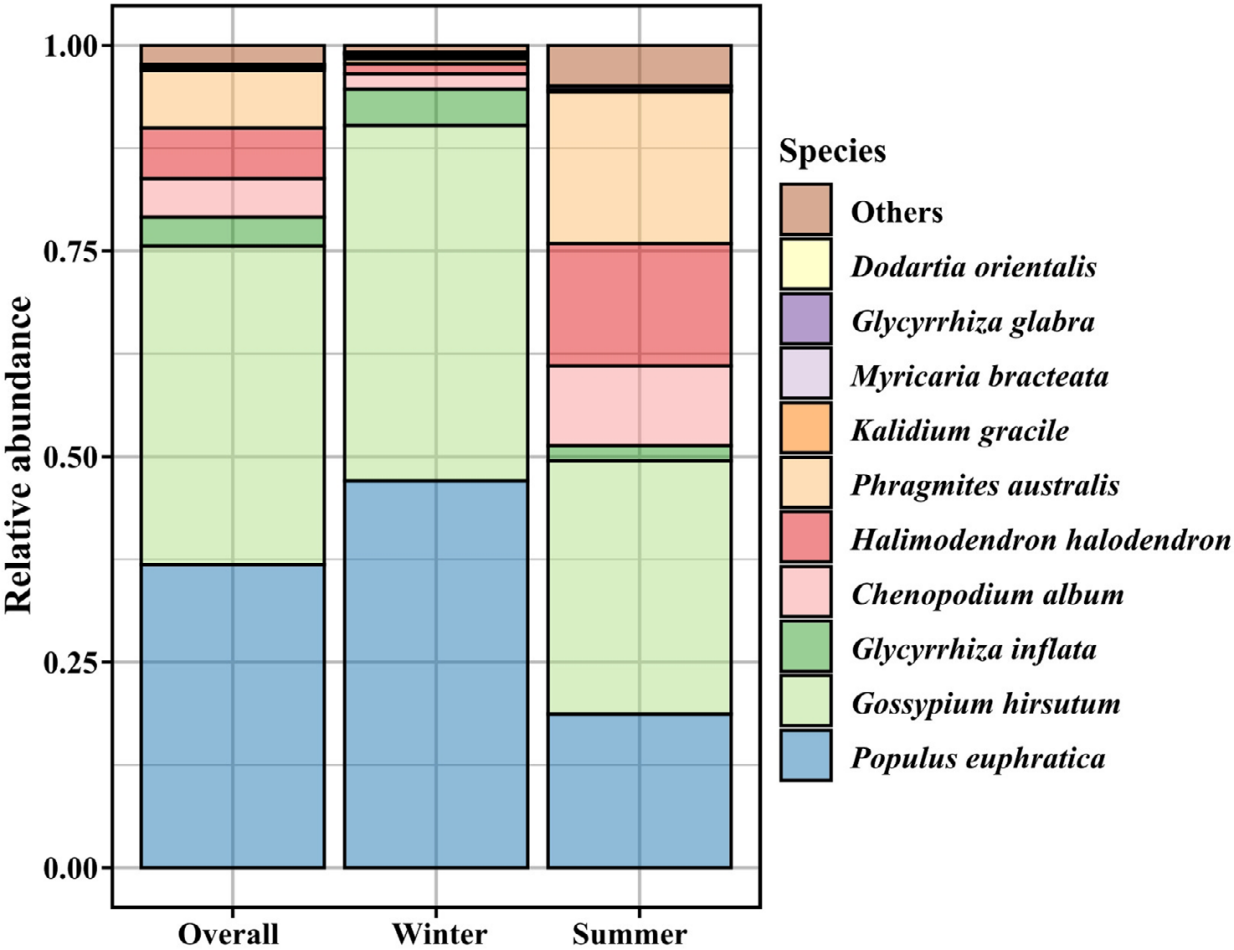
Relative abundance of diet composition at species levels of Tarim red deer using DNA macro‐barcoding technology in summer and winter.

**FIGURE 4:**
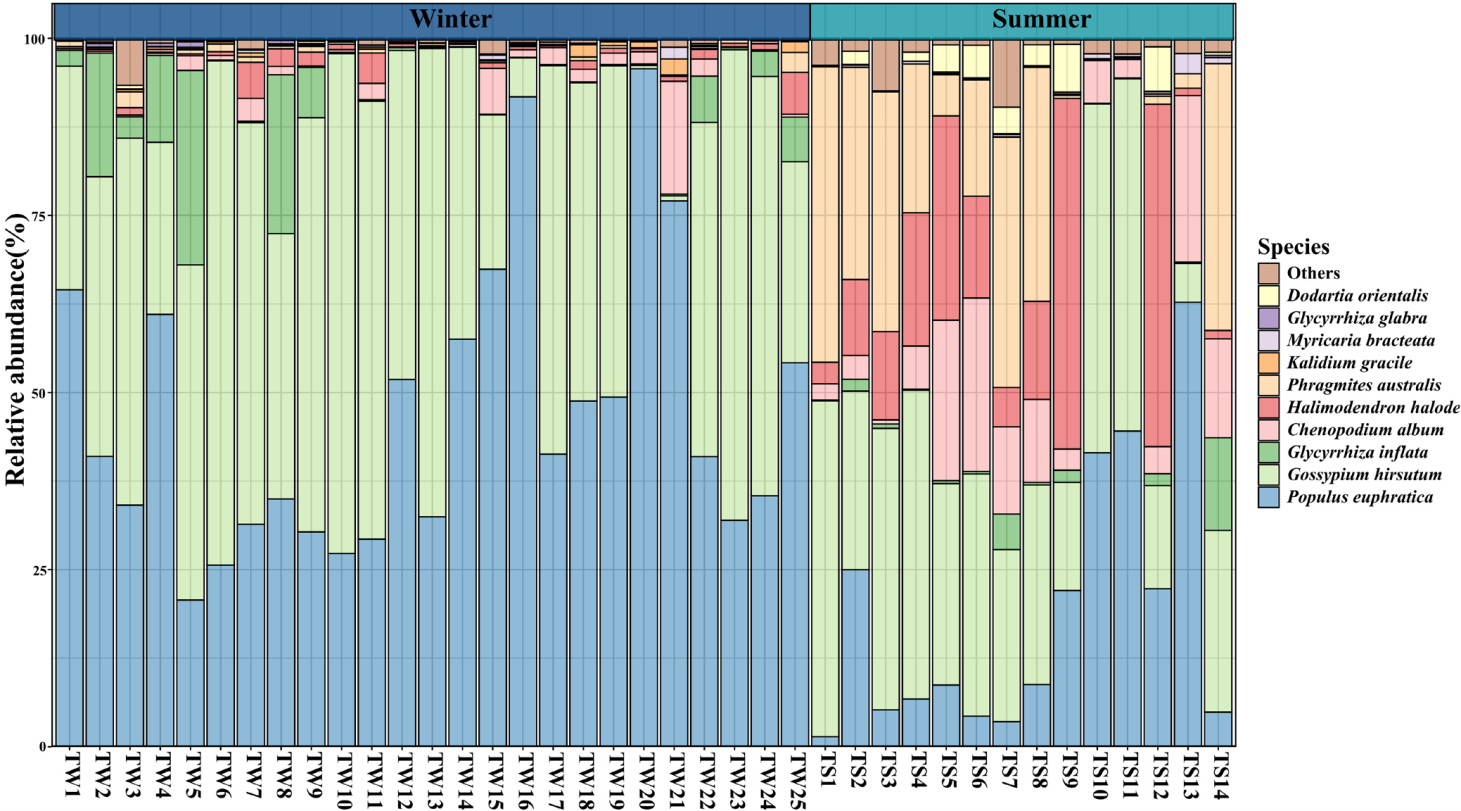
Relative abundance of diet composition consumed by Tarim red deer individual in summer and winter. TS refers to samples collected in summer, and TW refers to samples collected in winter.

Despite the important overlap of OTUs in winter and summer shown in Figure 2, diet composition is partly different in the two seasons (Figure 3). The OTUs clustering analysis indicated that Tarim red deer consumed a total of 50 plant species. Overall, the primary dietary components of the diet of Tarim red deer were cotton (38.75%) and Euphrates poplar (36.87%), while other species were found to be less significant, including common reed (7.00%), common salt tree (6.13%), goosefoot (*Chenopodium album*, 4.67%), and licorice (3.50%) (Figure 3). The summer diet was largely dominated by cotton (30.78%), but Euphrates poplar (18.70%), common reed (18.47%), common salt tree (14.86%), and goosefoot (9.74%) are present. In contrast, the winter diet was dominated by Euphrates poplar (47.04%) and cotton (43.22%) (Figure 3). During winter, dietary profiles showed limited inter‐individual divergence, with most samples characterized by a consistent dominance of Euphrates poplar and cotton; only a small number of samples were composed almost exclusively of poplar. In summer, however, inter‐individual variation increased markedly, with diets incorporating a broader spectrum of plant taxa (Figure 4).

**FIGURE 5:**
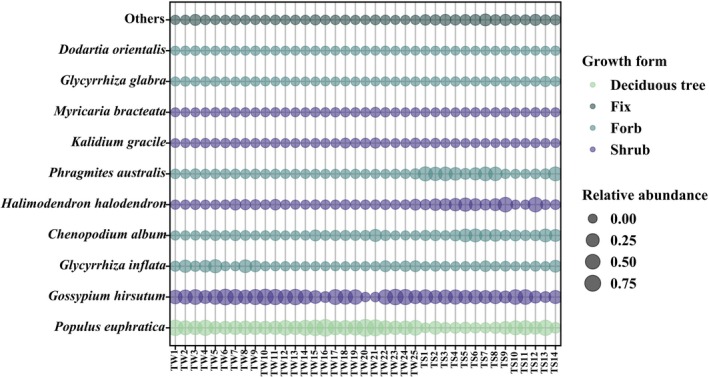
Relative abundance of the 10 most abundant plant species.

In winter the main food categories used by the Tarim red deer were deciduous trees (36.87%), shrubs (45.98%), non‐graminoid herbaceous (9.74%), and graminoid herbaceous (7.11%). In contrast, in summer shrubs (50.97%) exhibit the highest relative abundance, but the relative abundance of deciduous trees (17.19%), non‐graminoid herbaceous (14.92%), graminoid herbaceous (16.84%) were above 10% (Figure 5, Table S1).

### 3.3 Indicator Species Analysis

Indicator species analysis identified 24 plant taxa as significant indicators of summer foraging ecology. These comprised common reed, common salt tree, goosefoot, field sowthistle ( *Sonchus arvensis* ), Oriental dodartia (*Oriental Dodartia*), Venice mallow ( *Hibiscus trionum* ), sea buckthorn ( *Hippophae rhamnoides* ), Indian rushpea ( *Hoffmannseggia glauca* ), green bristlegrass ( *Setaria viridis* ), Mongolian calligonum (*Calligonum mongolicum*), Wilhelm's willow (*Salix wilhelmsiana*), Siberian elm ( *Ulmus pumila* ), weet wormwood ( *Artemisia annua* ), Chee reedgrass ( *Calamagrostis epigeios* ), field bindweed ( *Convolvulus arvensis* ), common wheat ( *Triticum aestivum* ), Siberian cocklebur ( *Xanthium sibiricum* ), creeping thistle ( *Cirsium arvense* ), jimsonweed ( *Datura stramonium* ), Indian lovegrass ( *Eragrostis pilosa* ), Tangut nitraria (*Nitraria tangutorum*), annual rabbitsfoot Grass ( *Polypogon monspeliensis* ), seepweed (*Suaeda salsa*), and corn ( *Zea mays* ), representing key functional groups within the dietary spectrum, and 4 indicator species including poplar, cotton, Hop dodder (*Cuscuta lupuliformis*), and slender branch saltwort in winter. In these species, 7 Gramineae herbs, 11 Non‐Gramineae herbs, 5 shrubs, and 1 deciduous trees were the indicator species in winter. 1 Non‐Gramineae herbs, 2 shrubs, and 1 deciduous trees were the indicator species in summer (Table 2).

**TABLE 2:** Indicator plant species and their relative abundance in the diet of Tarim red deer during summer and winter.

Season	Plant	Indicator		*p*	Mean relative abundance (%)		Growth form
		Summer	Winter		Summer	Winter	
Summer	*Ulmus pumila*	0.65	0.264	0.04	69.97	30.03	Deciduous trees
	*Phragmites australis*	0.97	0.03	0.001	96.96	3.04	Gramineae herbs
	*Setaria viridis*	0.964	0.035	0.001	96.38	3.62	Gramineae herbs
	*Calamagrostis epigeios*	0.954	0.044	0.025	95.39	4.61	Gramineae herbs
	*Triticum aestivum*	0.723	0.126	0.033	84.3	15.7	Gramineae herbs
	*Eragrostis pilosa*	0.85	0.007	0.012	99.12	0.88	Gramineae herbs
	*Polypogon monspeliensis*	0.496	0.001	0.002	99.21	0.79	Gramineae herbs
	*Zea mays*	0.774	0.043	0.001	90.26	9.74	Gramineae herbs
	*Chenopodium album*	0.842	0.158	0.006	84.17	15.83	Non‐Gramineae herbs
	*Sonchus arvensis*	0.943	0.057	0.008	94.26	5.74	Non‐Gramineae herbs
	*Dodartia orientalis*	0.959	0.041	0.004	95.87	4.13	Non‐Gramineae herbs
	*Hibiscus trionum*	0.627	0.373	0.048	62.73	37.27	Non‐Gramineae herbs
	*Hoffmannseggia glauca*	0.924	0.003	0.013	99.53	0.47	Non‐Gramineae herbs
	*Artemisia annua*	0.881	0.1	0.01	88.12	11.88	Non‐Gramineae herbs
	*Convolvulus arvensis*	0.877	0.079	0.003	87.66	12.34	Non‐Gramineae herbs
	*Xanthium sibiricum*	0.878	0.024	0.001	94.53	5.47	Non‐Gramineae herbs
	*Cirsium arvense*	0.692	0.015	0.005	96.89	3.11	Non‐Gramineae herbs
	*Datura stramonium*	0.501	0.015	0.001	87.72	12.28	Non‐Gramineae herbs
	*Suaeda salsa*	0.758	0.02	0.007	96.42	3.58	Non‐Gramineae herbs
	*Halimodendron halodendron*	0.923	0.077	0.001	92.28	7.72	Shrubs
	*Hippophae rhamnoides*	0.989	0.011	0.002	98.89	1.11	Shrubs
	*Calligonum mongolicum*	0.976	0.024	0.034	97.62	2.38	Shrubs
	*Salix wilhelmsiana*	0.815	0.185	0.006	81.51	18.49	Shrubs
	*Nitraria tangutorum*	0.457	0.003	0.003	91.46	8.54	Shrubs
Winter	*Gossypium hirsutum*	0.416	0.584	0.034	41.6	58.4	Shrubs
	*Kalidium gracile*	0.18	0.82	0.016	18.04	81.96	Shrubs
	*Cuscuta lupuliformis*	0.203	0.797	0.001	20.29	79.71	Non‐Gramineae herbs
	*Populus euphratica*	0.284	0.716	0.001	28.44	71.56	Deciduous trees

### 3.4 Alpha Diversity

**FIGURE 6:**
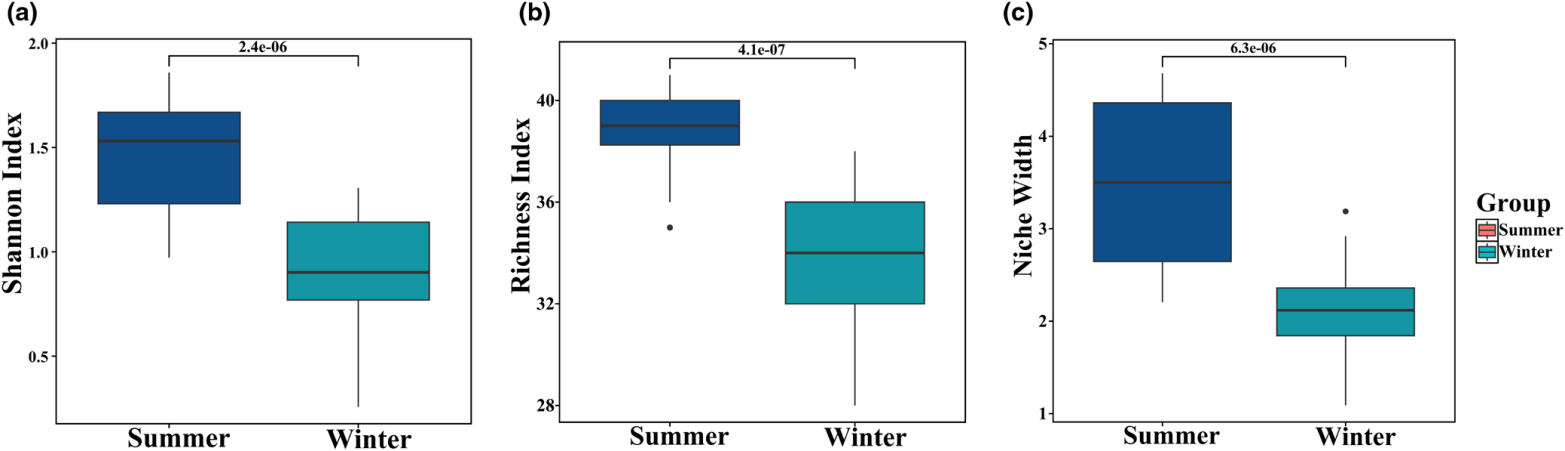
Differences in alpha diversity between summer and winter food composition of Tarim red deer. (a) Shannon index boxplot, (b) species richness boxplot, (c) Niche Width Boxplot.

Seasonal analysis of dietary diversity indices in the Tarim red deer revealed distinct patterns across multiple metrics. Dietary richness exhibited values of 34.08 ± 0.486 during winter and 38.642 ± 0.530 in summer. Niche breadth index registered 2.133 ± 0.097 in winter and 3.490 ± 0.240 in summer. Similarly, the Shannon index demonstrated values of 0.925 ± 0.052 for winter and 1.47 ± 0.073 for summer (Table S2). Shannon diversity index, richness index of food composition was significantly higher in summer than in winter (*p* < 0.05, *T*‐test, Figure 6a,b, Table S2). The dietary niche breadth results indicate that the dietary niche width in winter was significantly lower than in summer (*p* < 0.05, Wilcoxon‐test, Figure 6c, Table S3).

### 3.5 Beta Diversity

**FIGURE 7:**
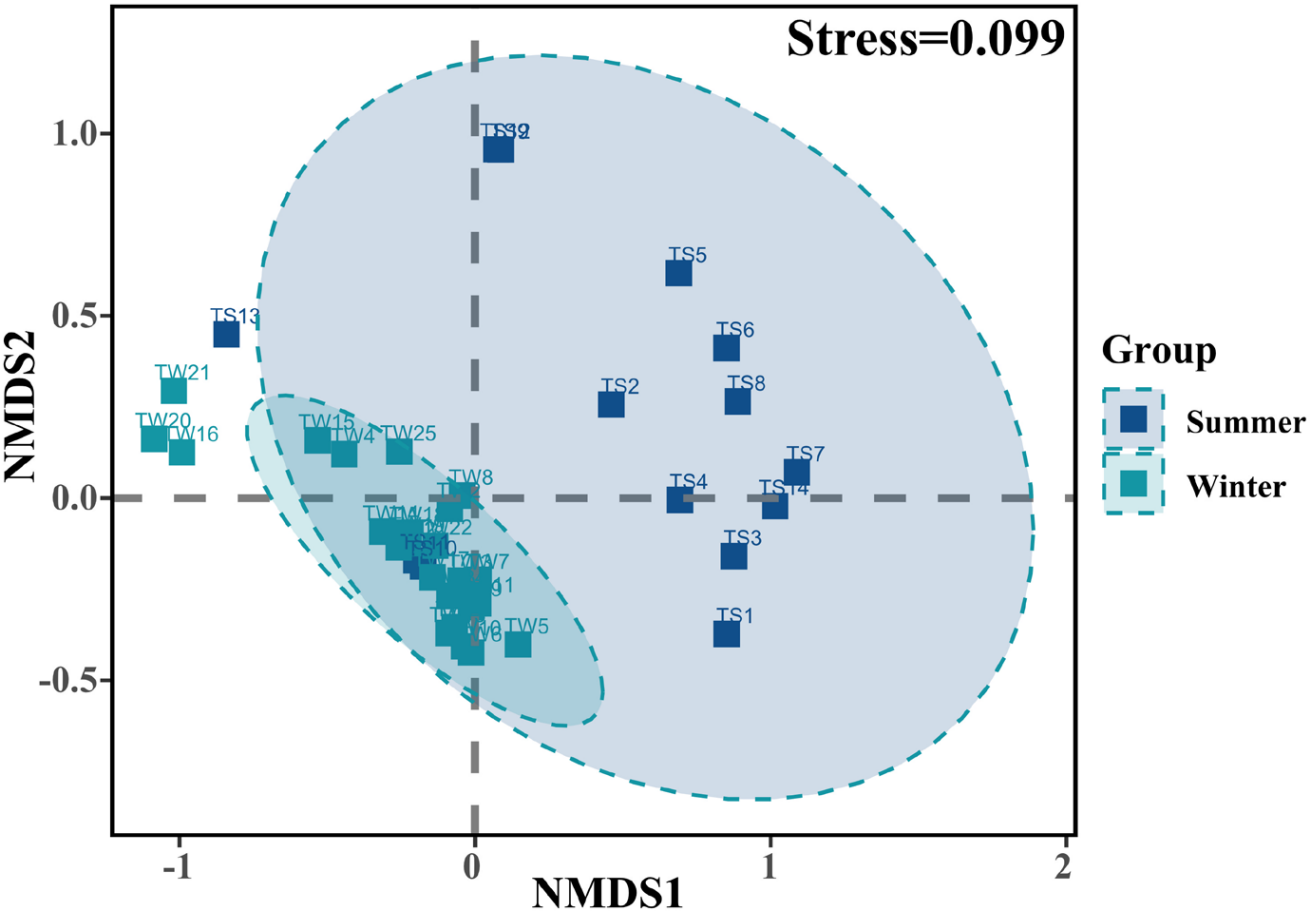
Analysis of Beta diversity in summer and winter food composition of Tarim red deer.

Clear differences in diet composition were observed between the seasons using NMDS plot (*R* ^2^ = 0.99, Stress = 0.099, Figure 7). The PERMANOVA test further demonstrated that the seasons accounted for approximately 27% of the compositional variance (pseudo‐*F* = 13.691; *p* < 0.05, *R* ^2^ = 0.27, Table S4). The ANOSIM test also indicated an appropriate grouping of both summer and winter feed (*R* = 0.5407, *p* < 0.05).

## 4 Discussion

Accordingly to available studies, Tarim red deer can be considered mixed or intermediate feed (Hofmann 1989). Our findings demonstrate that the Tarim red deer employs a facultative mixed foraging strategy, displaying significant dietary flexibility in response to seasonal variations. The observed seasonal shifts in diet composition reflect the temporal dynamics of resource availability within the study area. Furthermore, the dietary diversity patterns provide strong evidence that foraging behavior directly corresponds to plant resource fluctuations between summer and winter seasons, thereby substantiating the close relationship between diet selection and the phenological progression of plant communities in the poplar forest ecosystem of the Tarim River Basin. During the summer, the Tarim red deer consumed a wider range of herbaceous taxa, corresponding to the peak growth period of forbs and grasses. The landscape provides abundant and diverse vegetation, including young shoots, leaves, and herbaceous plants with high moisture and nutrient content, such as common reed and goosefoot. By contrast, the winter diet became dominated by woody plants, accompanied by a marked decline in herbaceous consumption (−24.29%). The proportion of deciduous woody species increased by 29.85% (primarily Euphrates Poplar) during winter, indicating a clear seasonal foraging shift toward browse‐dominated diets. The marked seasonal shift in dietary composition reflects the Tarim red deer's adaptive foraging strategy in response to changes in plant phenology and resource availability across the Tarim River Basin.

Similar seasonal dietary shifts toward woody browse and contractions in dietary breadth during the cold season have been reported in other cervid species inhabiting arid and temperate ecosystems. For instance, the Kashmir red deer (*Cervus hanglu hanglu*) shift from forbs to dicotyledonous browse during winter (Ahmad et al. 2016), while the sika deer display distinct seasonal foraging strategies, their summer diet is dominated by graminoids, forbs, and young leaves and buds of woody plants, whereas their winter diet shifts to a reliance on the leaves and bark of evergreen trees (Nakahama et al. 2021). Similarly, the wapiti (*Cervus canadensis xanthopygus*) consistently consumes a higher proportion of browse than graminoids across both summer and winter seasons. During summer, this pattern corresponds to the high density of woody vegetation within their activity ranges, while in winter, it represents an adaptive response to resource scarcity, as woody browse remains accessible above the snow cover (Furen et al. 2022). Likewise, sika deer in the Taohongling Sika Deer National Nature Reserve exhibit a 14.8% increase in shrub consumption during winter, a pattern attributed to the reduced availability of herbaceous plants in colder months (Wang et al. 2025). Fallow deer ( *Dama dama* ) also rely heavily on bramble ( *Rubus fruticosus* agg.) year‐round, with this genus forming a critical component of their winter diet; simultaneously, they exhibit a pronounced reduction in trophic niche breadth during winter, indicating a marked seasonal contraction in dietary diversity (Gresham et al. 2025). Collectively, these comparative findings support our observations in Tarim red deer, underscoring that increased reliance on woody plants and reduced dietary diversity are common adaptive responses among Cervids to winter resource limitation in temperate and arid regions. Our results suggest shrubs and poplar constituted the principal dietary components across both summer and winter seasons, demonstrating their pivotal role in the foraging ecology of the Tarim red deer. Shrubs remained the dominant dietary category year‐round, while Euphrates Poplar consistently represented the most consumed woody taxon, indicating strong dietary selectivity for these resources. The stable contribution of Euphrates poplar in the Tarim red deer diet reflects both its year‐round availability and its structural accessibility, as its shoots and fallen leaves provide reliable nutrition during times of scarcity.

Dietary diversity can be assessed using two complementary approaches: by measuring the number of distinct plant taxa consumed, or by assessing the phylogenetic breadth of foraging strategies when the focal species utilizes multiple plant lineages (Kartzinel and Pringle 2020). Precise dietary analyses inform the conservation and management of endangered ungulate populations (Bison et al. 2015). In high‐throughput sequencing‐based dietary studies, achieving taxonomic resolution at the genus or species level requires careful selection of DNA barcoding markers tailored to both the target taxonomic groups and the characteristics of fecal samples (Erickson et al. 2017, Ando et al. 2018, Spitzer et al. 2020). Among these, the *trnL* P6 loop region (primers trnL‐g/h) has become one of the most widely applied universal markers for detecting plant composition in herbivore feces (Ait Baamrane et al. 2012; Ando et al. 2018). In comparison to the field observations and fecal microscopic analyses conducted by Qiao et al. (2006), which identified 15 plant species in the diet of Tarim red deer—including common reed, licorice, and Euphrates poplar as year‐round dietary staples (Qiao et al. 2006). Our study identified a total of 50 plant species in the diet of Tarim red deer using *trnL*‐based DNA metabarcoding. These results demonstrate that the *trnL* barcoding approach is both appropriate and effective for characterizing the dietary composition of this species. Moreover, this methodology has strong potential for application in ecological investigations of other Tarim red deer populations across Central Asia.

It is particularly noteworthy that our data show that cotton constituted a substantial component of the Tarim red deer diet, accounting for 30.78% of total reads in summer and 43.22% in winter. Evidence of cotton field use was further confirmed through field investigations, including observations of fence‐jumping behavior and hair traces on barbed wire. This pattern indicates that the species has incorporated agricultural crops as predictable and nutritionally stable resources, particularly during periods of natural forage scarcity. Such behavioral adaptation parallels the foraging plasticity observed in European red deer ( *Cervus elaphus* ), which exploit crop fields as high‐energy, low‐risk food sources (Goldberg et al. 2020). In the Tarim River Basin, cotton fields function as an integrated “cotton‐field niche”, providing food, water, and safe movement corridors—especially after winter fence removal and when irrigation creates accessible water sources beneath thin ice layers. This resource system effectively supports Tarim red deer winter survival but simultaneously reflects an ecological dependence on anthropogenic landscapes.

Comparable patterns of crop use and human–wildlife conflict have been reported in other ungulate systems worldwide. Studies in Europe and South Asia reveal that the expansion of agricultural frontiers and habitat fragmentation have increased crop depredation by wild ungulates, particularly during resource‐scarce seasons. For instance, field‐based assessments in the Czech Republic showed that crop losses caused by wild boar and other large ungulates impose significant economic costs, regardless of the damage estimation method employed (Drimaj et al. 2023). Similarly, in India, cheetal ( *Axis axis* ) and sambar ( *Rusa unicolor* ) were identified as major crop raiders near forest edges, with damage peaking during early crop stages when fields were most palatable (Kumar et al. 2017). In Bhutan, ungulate abundance and crop damage were positively associated with proximity to croplands, indicating the attraction of agricultural areas as high‐energy foraging patches (Thinley et al. 2017). A pan‐European analysis further demonstrated that crop type, rather than ungulate density, is the principal driver of damage intensity, underscoring the decisive role of human land‐use decisions in shaping wildlife foraging behavior (Widén et al. 2023). Collectively, these studies emphasize that the increased consumption of crops by Tarim red deer reflects a broader ecological trend: large herbivores increasingly integrate agricultural resources into their foraging strategies in response to altered foodscapes and shrinking natural habitats. However, this behavioral adaptation entails ecological and physiological risks. Cotton plants contain gossypol, a polyphenolic compound known for its potential toxicity to ruminants (Tian et al. 2016). Although mature individuals may partially detoxify or bind gossypol through ruminal processes (Hongli and YuLiang 2005; Jun et al. 2008), excessive intake can lead to anemia, reduced fertility, and digestive disturbances. More critically, sustained dependence on croplands increases exposure to pesticides, human disturbance, and retaliatory persecution, further exacerbating conservation challenges. Similar risks have been observed in Europe, where high ungulate densities near farmland have intensified conflicts, prompting costly and often ineffective mitigation measures (Reimoser and Putman 2011). These converging lines of evidence suggest that the Tarim red deer's increasing reliance on agricultural resources reflects not ecological success, but an adaptive response to habitat degradation and nutritional stress within its native Euphrates poplar forests.

Effective mitigation must therefore move beyond physical exclusion measures such as fencing, which merely displace conflict rather than resolving it. Instead, conservation strategies should adopt an integrated landscape approach that balances agricultural production with wildlife needs. Restoring the structural complexity of Euphrates Poplar riparian forests, promoting understory herbaceous regeneration, and ensuring ecological water allocations are essential to rebuilding the natural forage base. At the same time, management strategies proven effective elsewhere—such as modifying crop types to less palatable varieties, creating buffer zones, or establishing deterrent planting schemes (Widén et al. 2023)—should be adapted to the Tarim context. Supplementary feeding stations and targeted habitat restoration could redirect deer from croplands during critical periods, reducing economic losses while maintaining population stability. Collectively, these measures will help mitigate human–wildlife conflict, restore the ecological resilience of the Tarim riparian system, and ensure the long‐term conservation of Tarim red deer.

In summary, our findings demonstrate that the Tarim red deer exhibits pronounced seasonal dietary flexibility (supporting H1), persistent dependence on native woody vegetation (supporting H2), and increasing adaptation to agricultural landscapes (supporting H3). While this plasticity facilitates short‐term persistence in human‐dominated environments, it masks deeper ecological degradation and long‐term population risks. The species' dual reliance on poplar and cotton illustrates a precarious balance between native and anthropogenic resources—neither independently sufficient for population stability. Sustainable conservation of the Tarim red deer will therefore require a coordinated strategy integrating riparian forest restoration, ecological water management, and proactive conflict mitigation. Reinstating the functional connectivity and productivity of native ecosystems is essential to reducing agricultural dependence and re‐establishing the ecological integrity of the Tarim River Basin.

## 5 Conservation Implications

Our findings provide important insights for the conservation and management of Tarim red deer inhabiting in the Euphrates poplar riparian ecosystem in the Tarim River Basin. Euphrates poplar and Cotton have become the primary and stable food sources for the Tarim red deer, yet the increasing use of cotton fields—up to 43.22% of winter intake—signals growing dependence on anthropogenic landscapes. This behavioral shift mitigates food shortages but exposes the population to potential gossypol toxicity and intensifies human–wildlife conflict. Therefore, future research should prioritize investigating the macronutrient composition of available forage and elucidating the corresponding nutritional strategies adopted by Tarim red deer. Particular attention should be given to the potential ecological and physiological risks, including the accumulation of pesticide residues and the metabolic impacts of gossypol exposure. From a habitat management perspective, conservation efforts should focus on restoring Euphrates poplar riparian forests and enhancing the productivity of understory vegetation through improved ecological water management. Such measures would strengthen the natural forage base, thereby reducing the species' dependence on agricultural crops. Ultimately, these integrated ecological and management interventions will promote the long‐term coexistence of Tarim sika deer populations and local agricultural communities throughout the Tarim River Basin.

## Author Contributions

**Cheng Qi:** conceptualization (equal), data curation (equal), formal analysis (equal), investigation (equal), methodology (equal), resources (equal), software (lead), supervision (supporting), validation (equal), visualization (equal), writing – original draft (equal), writing – review and editing (equal). **Xiangzhou Tian:** investigation (equal). **Yuanfan Cui:** writing – original draft (equal), writing – review and editing (supporting). **Linyin Zhu:** investigation (equal), resources (supporting). **Stefano Focardi:** writing – original draft (supporting), writing – review and editing (supporting). **Linqiang Zhong:** conceptualization (equal), data curation (equal), formal analysis (equal), funding acquisition (lead), investigation (equal), methodology (equal), project administration (lead), resources (equal), software (equal), supervision (lead), validation (equal), visualization (equal), writing – original draft (equal), writing – review and editing (equal).

## Funding

This work was supported by the Natural Science Foundation of Xinjiang Uygur Autonomous Region (2022D01C657) and Scientific Research Initiation Program of Xinjiang University (620321053).

## Conflicts of Interest

The authors declare no conflicts of interest.

## Supporting information


**Data S1:** ece372757‐sup‐0001‐Supinfo.docx.

**Data S2:** ece372757‐sup‐0002‐Supinfo1.zip.

## Data Availability

The data that support the findings of this study are openly available.
