# Supplementary material for: Seasonal Dietary Patterns of Tarim Red Deer (Cervus hanglu yarkandensis) Revealed by the trnL Sequencing Approach in the Tarim River Basin (Xinjiang, China)
Source: Ecol Evol. 2025 Dec 23;15(12):e72757. doi: 10.1002/ece3.72757 (PMC12724009; doi:10.1002/ece3.72757)
Supplement: Supplementary file 1 — Data S1: ece372757‐sup‐0001‐Supinfo.docx. [file ECE3-15-e72757-s002.docx]

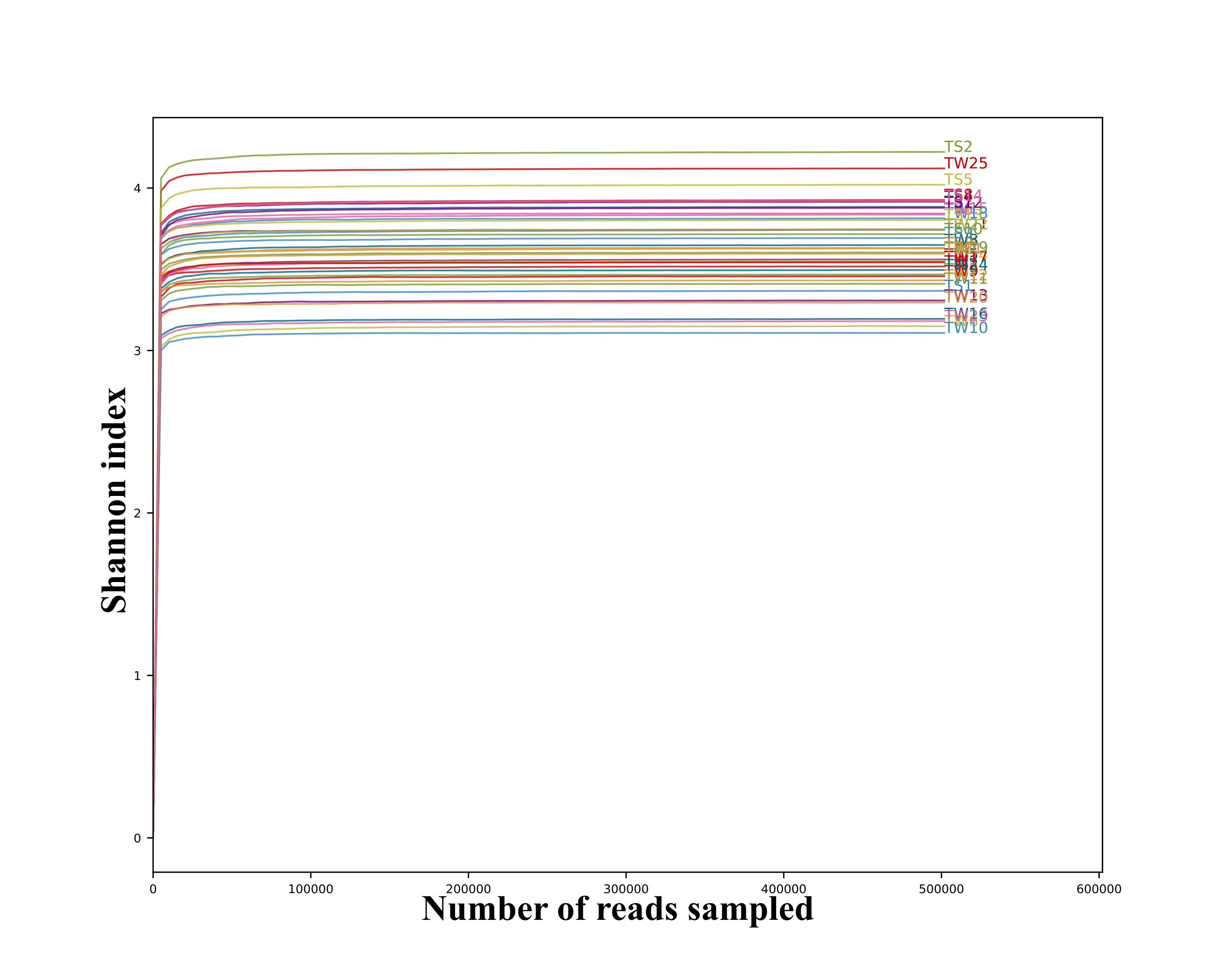


FIGURE S1 Rarefaction curves of Shannon–Wiener diversity in this study.


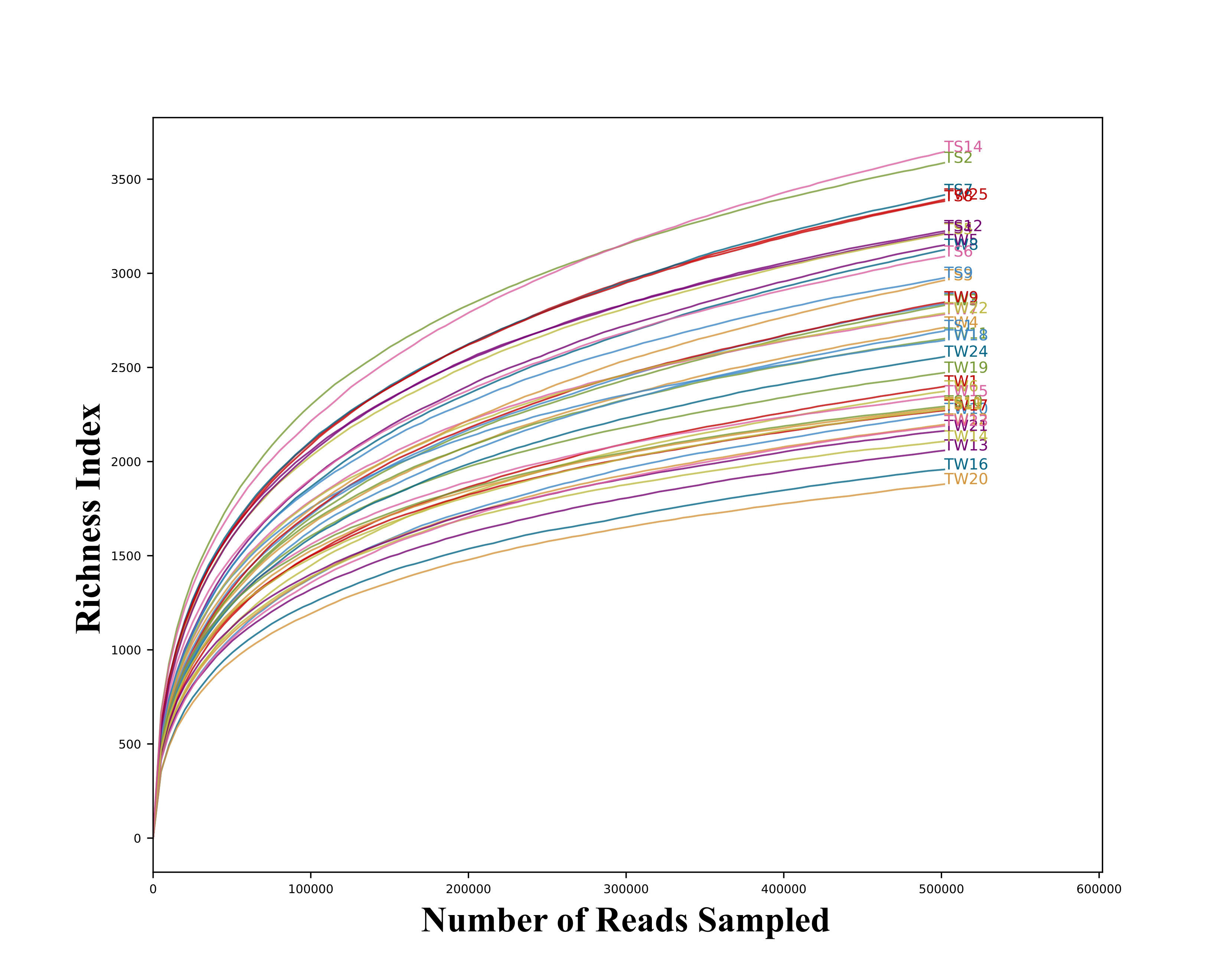


FIGURE S2 species richness for the diet of Tarim red deer in this study

TABLE S1 Percentage of food categories in the diet of Tarim red deer.

| **Growth form** | **Overall (%)** | **Winter(%)** | **Summer(%)** |
| --- | --- | --- | --- |
| Deciduous trees | 36.87 | 47.04 | 17.19 |
| Shrubs | 45.98 | 45.08 | 50.97 |
| Non-graminoid herbaceous plants | 9.74 | 6.87 | 14.92 |
| Graminoid herbaceous plants | 7.11 | 0.60 | 16.84 |
| Unknown | 0.29 | 0.40 | 0.08 |

TABLE S2 Alpha diversity of plant species in the diet of Tarim red deer.

| **Sample ID** | **Shannon** | **Richness** | **Niche Breadth** |
| --- | --- | --- | --- |
| TW1 | 0.8318570 | 32 | 1.937256 |
| TW2 | 1.1660730 | 36 | 2.822185 |
| TW3 | 1.2224484 | 36 | 2.564488 |
| TW4 | 1.0484971 | 36 | 2.239063 |
| TW5 | 1.2593257 | 35 | 2.919664 |
| TW6 | 0.7630401 | 31 | 1.747145 |
| TW7 | 1.1418684 | 35 | 2.359841 |
| TW8 | 1.3065817 | 33 | 3.187411 |
| TW9 | 1.0645425 | 32 | 2.276753 |
| TW10 | 0.7254240 | 31 | 1.746759 |
| TW11 | 1.0135798 | 32 | 2.126103 |
| TW12 | 0.8041639 | 36 | 2.063891 |
| TW13 | 0.7284741 | 34 | 1.843919 |
| TW14 | 0.7687429 | 37 | 1.997206 |
| TW15 | 0.9977807 | 37 | 1.974665 |
| TW16 | 0.3954429 | 28 | 1.183750 |
| TW17 | 0.8810862 | 36 | 2.118955 |
| TW18 | 1.0041438 | 38 | 2.268974 |
| TW19 | 0.9008984 | 34 | 2.162162 |
| TW20 | 0.2573053 | 31 | 1.091464 |
| TW21 | 0.8164992 | 33 | 1.613014 |
| TW22 | 1.1468053 | 33 | 2.529783 |
| TW23 | 0.7308930 | 36 | 1.839821 |
| TW24 | 0.9014951 | 34 | 2.095814 |
| TW25 | 1.2536187 | 36 | 2.612631 |
| TW-MEAN | 0.9252235 | 34.08 | 2.132909 |
| TW-SE | 0.05153405 | 0.4862098 | 0.09741706 |
| TS1 | 1.162313 | 41 | 2.494066 |
| TS2 | 1.689477 | 39 | 4.365743 |
| TS3 | 1.474531 | 36 | 3.401847 |
| TS4 | 1.560812 | 40 | 3.597302 |
| TS5 | 1.668821 | 40 | 4.389015 |
| TS6 | 1.672476 | 39 | 4.380186 |
| TS7 | 1.858736 | 39 | 4.682054 |
| TS8 | 1.666725 | 39 | 4.347775 |
| TS9 | 1.433138 | 35 | 3.099362 |
| TS10 | 1.054590 | 35 | 2.389233 |
| TS11 | 0.972375 | 41 | 2.238163 |
| TS12 | 1.500924 | 39 | 3.221187 |
| TS13 | 1.151831 | 38 | 2.205584 |
| TS14 | 1.659998 | 40 | 4.053374 |
| TS- MEAN | 1.466196 | 38.64286 | 3.490349 |
| TS-SE | 0.07352075 | 0.5304688 | 0.2402641 |
| MEAN | 1.119419 | 35.71795 | 2.620195 |
| SE | 0.05924004 | 0.5063013 | 0.1486259 |

TABLE S3 Analysis of variance for seasonal variation in alpha diversity indices.

|  | **Normality Test(*p*)** | **Levene Test(*p*)** | **t/W** | **df** | ***p*** |
| --- | --- | --- | --- | --- | --- |
| Shannon | 0.3876 | 0.6889 | 6.1437 | 37 | 4.013e-07 |
| Richness | 0.3157 | 0.2567 | 5.9841 | 37 | 6.598e-07 |
| Niche width | 0.003607 | 0.001427 | 317(W) |  | 6.321e-06 |

TABLE S4 PERMANOVA results for seasonal variation in food composition of Tarim red deer.

|  | **df** | **F.model** | **R^2^** | **F** | **Pr(>F)** |
| --- | --- | --- | --- | --- | --- |
| Season | 1 | 1.3179 | 0.27009 | 13.691 | 0.01 |
| Residual | 37 | 3.5614 | 0.75991 |  |  |
| Total | 38 | 4.8793 | 1 |  |  |
